# Oxygen, Gastrin-Releasing Peptide, and Pediatric Lung Disease: Life in the Balance

**DOI:** 10.3389/fped.2014.00072

**Published:** 2014-07-18

**Authors:** Mary E. Sunday

**Affiliations:** ^1^Department of Pathology, Duke University Medical Center, Durham, NC, USA

**Keywords:** oxygen-sensing cells, pulmonary neuroendocrine cells, pulmonary fibrosis, radiation injury, bronchopulmonary dysplasia, macrophages, fibroblasts

## Abstract

Excessive oxygen (O_2_) can cause tissue injury, scarring, aging, and even death. Our laboratory is studying O_2_-sensing pulmonary neuroendocrine cells (PNECs) and the PNEC-derived product gastrin-releasing peptide (GRP). Reactive oxygen species (ROS) generated from exposure to hyperoxia, ozone, or ionizing radiation (RT) can induce PNEC degranulation and GRP secretion. PNEC degranulation is also induced by hypoxia, and effects of hypoxia are mediated by free radicals. We have determined that excessive GRP leads to lung injury with acute and chronic inflammation, leading to pulmonary fibrosis (PF), triggered via ROS exposure or by directly treating mice with exogenous GRP. In animal models, GRP-blockade abrogates lung injury, inflammation, and fibrosis. The optimal time frame for GRP-blockade and the key target cell types remain to be determined. The concept of GRP as a mediator of ROS-induced tissue damage represents a paradigm shift about how O_2_ can cause injury, inflammation, and fibrosis. The host PNEC response *in vivo* may depend on individual ROS sensing mechanisms and subsequent GRP secretion. Ongoing scientific and clinical investigations promise to further clarify the molecular pathways and clinical relevance of GRP in the pathogenesis of diverse pediatric lung diseases.

## Introduction

Oxygen (O_2_) is essential for life. In aerobic animals, the lung evolved as a critical organ for gas exchange permitting species to move from water to land. Lungs are exposed to all the elements: air, earth, water, and fire/radiation. Homeostasis and health represent a natural equilibrium between opposing forces. Disease results when there is an imbalance between environmental exposures and host defense. Individual responses to diverse challenges can vary due to genetic factors.

Life hinges on a delicate balance. Too much or too little heat, humidity, or O_2_ can be lethal. Although O_2_ is essential for life, too much O_2_ can lead to tissue injury, fibrosis, senescence, and death (1–4). For several decades my research has focused on O_2_-sensing pulmonary neuroendocrine cells (PNECs) and their product gastrin-releasing peptide (GRP), a mammalian homolog of amphibian bombesin (5). GRP secretion can be induced by reactive oxygen species (ROS) from exposure to hyperoxia (6), ozone (7), or ionizing radiation (RT) (8). Furthermore, PNEC degranulation is known to be induced by hypoxia (9), which is also associated with increased ROS levels (10).

In the current review, I will introduce background information about PNECs as O_2_-sensing cells. The discussion will then summarize the highlights of over 25 years of work from my laboratory regarding the role of GRP in lung development and postnatal lung diseases, especially bronchopulmonary dysplasia (BPD). Cumulatively, these studies provide the foundation for future exploration of how GRP could mediate lung injury including acute and chronic inflammation and pulmonary fibrosis (PF) (7, 8, 11).

## Oxygen-Sensing Cells: Pulmonary Neuroendocrine Cells

**Figure 1:**
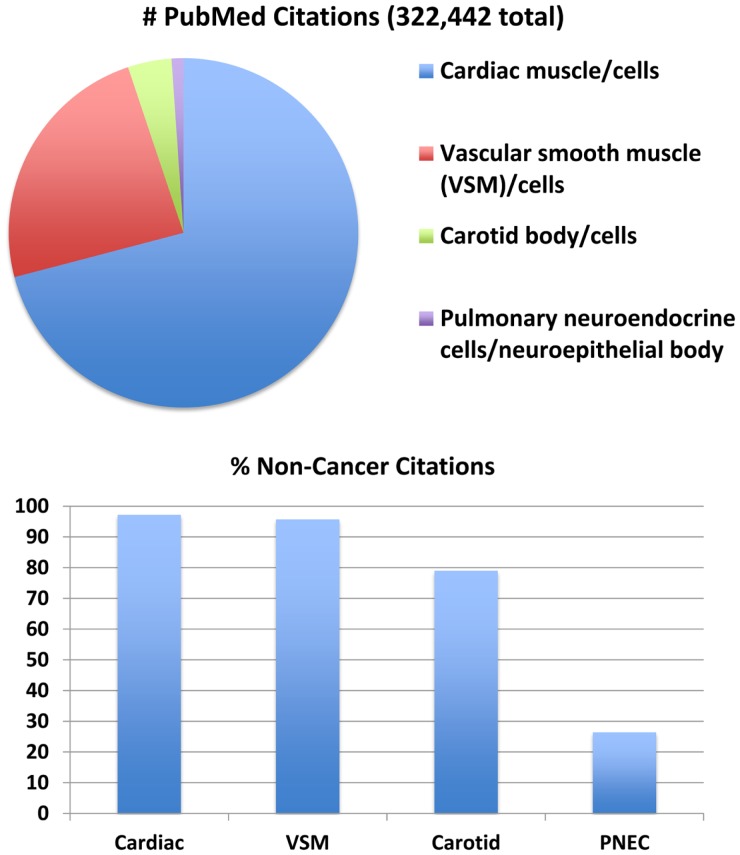
**Major subsets of O_2_-sensing cells: proportions of the total number of 322,442 publications per subgroup**. Top panel: relative numbers of citations per subgroup in July 2014. This comparison addresses four major subgroups of O_2_-sensing cells: cardiac muscle/cardiomyocytes, vascular smooth muscle (VSM)/myocytes, carotid body/glomus cells, and pulmonary neuroendocrine cells (PNECs)/neuroepithelial bodies. Actual numbers are given in the text. Lower panel: when citations including the keyword “cancer” are excluded, the number of citations is decreased per subset by 3–21% for cardiac muscle, vascular smooth muscle, and carotid body, but is decreased 74% for PNECs. (Mary E. Sunday, original unpublished data)

O_2_-sensing cells are important regulators of vascular tone and cardiac function. Historically, most research about O_2_-sensing cell biology and physiology has been focused on cardiomyocytes (12), vascular smooth muscle cells (13), and carotid body cells (glomus cells) (14), although interest in PNEC biology is growing (15). Much has been written about all of these cells (Figure 1) and their collective tissues, with numbers of PubMed citations on July 5, 2014 as follows: 228,676 for cardiomyocyte(s), cardiac muscle cell(s), or cardiac muscle (cells vs. tissue = 61,228 vs. 213,615); 77,151 for vascular smooth muscle cell(s), vascular smooth myocyte(s), or vascular smooth muscle (cells vs. tissue = 53,567 vs. 72,628); 13,068 for carotid body cell(s), glomus cells(s) or carotid body (cells vs. tissue = 6,462 vs. 11,987). However, relatively little is known about PNECs or their clusters in pulmonary epithelium, called neuroepithelial bodies (NEBs): 3,547 total citations, representing 3048 for PNECs and/or 624 for NEB(s). If cancer is excluded from the search for the cardiac, vascular, or carotid cells or tissues, the numbers drop modestly (Figure 1, lower panel) with the percentage of non-cancer citations: cardiac muscle or myocytes and vascular smooth muscle or myocytes, and 79–80% for carotid body or glomus cells. In contrast, the numbers of non-cancer citations for PNECs is only 26%, providing objective evidence that PNEC research has been largely focused on lung cancer, especially small cell carcinoma of the lung, a highly malignant cancer apparently derived from PNECs (16). Although a PubMed search for NEBs yielded only 624 citations, 508 (81%) of these are not related to cancer, possibly because PNEC-derived cancers do not develop as normal, slow-growing, innervated, and organoid NEBs (17). Of note, postnatally and in adults, NEBs represent an important stem cell niche involved in lung injury/repair as well as lung carcinogenesis (17–21). The biology of non-neoplastic/homeostatic PNEC responses to environmental challenges has been relatively under-explored. The low number of cancer-related publications is also likely due in part to challenges in culturing normal PNECs or NEBs, which have a low rate of cell proliferation both *in vitro* (22, 23) and *in vivo* (24), although PNEC proliferation can occur *in vivo* following acute injury (25).

PNECs were first identified in the lung by Feyrter as part of a diffuse epithelial endocrine system (26, 27). Studying airway epithelium of human newborn lung, Lauweryns later identified clusters of similar amine-producing cells, which he called “NEBs,” containing dense-core neurosecretory vesicles (DCV) (28). He investigated physiological responses of PNECs to altered O_2_ and CO_2_ levels in a series of seminal experiments (9, 29–31). He first studied hypoxia- or hypercarbia-induced exocytosis of DCV from NEBs (9). Second, by using cross-circulation studies in rabbits, he observed that airway hypoxia but not hypoxemia induced exocytosis of DCV from NEBs (30). He postulated that NEB react to the composition of inhaled air and by releasing serotonin or peptides could produce a local vasoconstriction and/or bronchoconstriction in hypoxically aerated lung areas, thus enabling intrapulmonary regulation of the V/Q ratio (30). Innervation of single PNECs and NEBs is extensive in newborn rabbits (32), consisting predominantly of vagal afferent sensory nerves (15, 33). Although the function of NEB innervation remains unclear, evidence suggests a role in the generation of dyspnea (34).

Investigating how PNECs sense hypoxia, Cutz et al. carried out patch-clamp analysis of intact NEBs stained with a vital dye. They found the key players in rabbit and human lung are a membrane-bound O_2_-binding NADPH oxidase coupled to an H_2_O_2_-sensitive K^+^ channel protein (35, 36), later confirmed in knockout mice as Nox2 (37). Although NEBs express multiple NADPH oxidases and diverse voltage-gated potassium channels (Kv) and tandem pore acid-sensing K^+^ channels (TASK) (38), there is molecular complex formation between NOX2 (gp91 phox) and Kv but not TASK1. This observation implicates NOX2/Kv as the major O_2_ sensor complex in PNECs (39, 40).

## Gastrin-Releasing Peptide during Physiological Hypoxia and Physiological Hyperoxia

Ernest Cutz is a pediatric pathologist who has carried out much of the seminal work on PNECs and GRP in pediatric lung diseases (17, 41). Writing a chapter together, we explored temporal and spatial expression of GRP expression during perinatal physiological processes versus postnatal disease states (42). This dichotomy can be viewed as functions of GRP in fetal lung development and perinatal transitioning (physiological hypoxia and physiological hyperoxia) versus GRP mediating pathological responses to sustained hyperoxic exposure, such as BPD.

*In utero* development can be considered a state of “physiological hypoxia.” Peak PNECs occur during the canalicular stage of development (at midgestation in primates and during late gestation in rodents), during which the foundation of the pulmonary capillary bed is established. At term, the umbilical artery pO_2_ is ~16 mm Hg (~24% O_2_ saturation), and umbilical vein pO_2_ is ~27 mm Hg (~55% O_2_ saturation), in contrast to postnatal arterial pO_2_ of ~100 mm Hg with O_2_ saturation >90% for term infants on room air (43).

Peak GRP mRNA levels are present in human fetal lung at midgestation (44), in the setting of physiological hypoxia (43). GRP (also known as bombesin, bombesin-like peptide or BLP) is initially synthesized as a 138–148 amino acid pro-hormone composed of three isoforms (45). These are all cleaved at methionine #27. This Met becomes the carboxy terminus of GRP that must be amidated to form the bioactive GRP peptide with GRP (14–27) amino acid sequence of – Met-Tyr-Pro-Arg-Gly-Asn-His-Trp-Ala-Val-Lys-His-Leu-Met-NH_2_.

Intrigued by this prenatal abundance of GRP gene expression, my laboratory began testing whether GRP alters fetal lung development. Our approach has focused on mouse, human, and baboon fetal lung organ cultures, and developing mice *in utero*. Cumulatively, we have determined that GRP or its amphibian homolog bombesin can promote widespread cell proliferation and accelerated differentiation of type 2 pneumocytes and PNECs (46–50). These observations were later confirmed by Fraslon and Bourbon in France (51) and Asokananthan and Cake in Australia (52), with the additional observation of GRP-induced surfactant secretion (52). We also determined that bombesin and a related frog peptide, Leu^8^-phyllolitorin, promote branching morphogenesis and cell proliferation in embryonic mouse lung buds (53).

In contrast to *in utero* development, postnatal adaptation is often referred to as “physiological hyperoxia” in recognition of the sudden change in O_2_ levels in the infant from ~27 mm Hg *in utero* to 100 mm Hg on room air (43). Room air is essentially hyperoxic to the newborn lungs. It has been recognized since the 1950s that postnatal lung development in premature infants is a unique medical situation, as first defined in pioneering work by Mel Avery that led to the discovery that respiratory distress syndrome (RDS) is due to a deficiency of surfactant. Consequently, preterm infants cannot readily expand their lungs with air to allow breathing (54). Before the arrival of surfactant therapy, premature infants often needed high levels of O_2_ therapy to survive. New challenges arose because prematurity is also associated with inadequate antioxidant defenses (55). Chronic lung disease of newborns, called BPD (56), was linked to O_2_ therapy, a mainstay of treatment for premature infants (57). The severity of BPD has decreased thanks to surfactant therapy and modern medical management such as low-barotrauma high-frequency ventilation and CPAP (58, 59). Despite improved medical care, the incidence of BPD has paradoxically increased or remains unchanged, which is puzzling regardless of how BPD is defined (57, 60, 61).

## BPD: Neuroendocrine Cells and Gastrin-Releasing Peptide

Bronchopulmonary dysplasia remains a major cause of morbidity and mortality in very low birth weight infants with gestational age <28 weeks (60, 62). BPD is associated with persistent respiratory morbidity including increased hospital admissions for respiratory distress, bronchiolitis, status asthmaticus, and pneumonia (59). BPD is also associated with other complications including pulmonary hypertension, systemic hypertension, intraventricular hemorrhage, periventricular leukomalacia, neurocognitive delay, and cerebral palsy (62–65).

Early prediction of BPD has proven challenging. Relative numbers of GRP-positive PNECs normally decrease over the first postnatal months, and are markedly decreased in premature infants dying of RDS at postnatal day (PND) 1–7, thought to reflect PNEC degranulation (66). In contrast, PNECs are increased in bronchioles of infants dying with BPD at 2 weeks to 6 months of age (66). We hypothesized that elevated urine GRP levels precede BPD. One hundred thirty-two infants born at 28-weeks gestation or less, were studied. Urine GRP levels, determined by radioimmunoassay, were normalized for creatinine. BPD was defined as O_2_ dependence at 36 weeks post-menstrual age. Consistent with the increased number of PNECs, urine GRP was also elevated in a first urine sample at PND 1–5 in ≤28-week gestation infants who later developed BPD (67). GRP is excreted as a stable peptide in the urine; urine GRP levels are positively correlated with bronchoalveolar lavage (BAL) GRP levels (68). In the analysis by Anne Cullen (now Anne Cullen Twomey), a first urine specimen with GRP level greater than 20,000 pg/mg creatinine between PND 1–5 occurred among 54% of the infants who later developed BPD (*p* < 0.001), versus 10% among non-BPD infants (specificity 90%). Multivariable logistic regression analyses demonstrated that elevated urine GRP levels were associated with a 10-fold increased risk of BPD (*p* < 0.001) after adjusting for all confounding factors. Furthermore, urine GRP elevation occurs in parallel with markedly increased levels of GRP mRNA in newborn baboon lung (69). Utilizing urine GRP for screening might permit early therapeutic interventions to reduce disease progression and could provide a target for new preventive therapies.

We tested the hypothesis that GRP is linked to the pathogenesis of BPD through analysis of two baboon models of BPD: hyperoxia (140-day-old animals [∼32 weeks human gestational equivalent] given 100% O_2_ for 10 days, vs. non-BPD 140-day-old animals given PRN O_2_) and barotrauma (125-day-old animals [∼26 weeks human gestational equivalent] given PRN O_2_ for 14 days) in collaboration with Jackie Coalson and the NIH Program in BPD (70–72). In both BPD models, GRP was elevated at 24–72 h after birth. This GRP elevation was closely correlated with impaired respiratory function with increased oxygenation index, and also arrested alveolar number with alveolar wall thickening, decreased secondary alveolar septa, and blunted capillary tubulogenesis (69, 73). Remarkably, postnatal inhibition of GRP with a blocking anti-GRP antibody prevented the functional and histological changes of BPD in these animal models (69, 73). These observations suggest that GRP could be an important therapeutic target to decrease BPD prevalence and later pulmonary morbidity.

## Oxidative Stress, Neuroendocrine Cells, and Gastrin-Releasing Peptide

PNEC hyperplasia occurs in weanling rat lungs in response to cigarette smoke (74) or hyperoxia (75). Elevated GRP has been associated with oxidative stress in humans including cystic fibrosis (CF) patients (76), asymptomatic smokers (68, 77), and patients with chronic obstructive pulmonary disease (78).

ROS, also known as oxygen free radicals, have been implicated in the pathogenesis of BPD. In the hyperoxic baboon model of BPD, inhibition of oxidative stress using a catalytically active metalloporphyrin (AEOL10113) decreased the number of PNEC cells, decreased GRP levels, and diminished BPD severity pathologically (6). The antioxidant not only decreased PNECs, but abrogated parenchymal mast cells and eosinophils (6). Subsequent work determined a direct link between GRP and mast cell accumulation (79). Despite the epidemiologic evidence that oxidative stress is linked to risk for BPD, this knowledge has not yet been translated into validated biomarkers for disease, or into mechanism-specific therapies to mitigate BPD morbidity.

Notably, several urine biomarkers of oxidative stress have been shown to be elevated in BPD in published clinical studies: F_2_-isoprostane (80, 81), 8-hydroxydeoxyguanosine (82, 83), and allantoin (84). F_2_-isoprostanes are increased in term infants ventilated with FiO_2_ of 1.0 for severe pulmonary disease due to meconium aspiration, neonatal pneumonia, or primary pulmonary hypertension (85) or in preterm infants with BPD. 8-hydroxy-2′-deoxyguanosine is an established marker of *in vitro* and *in vivo* oxidative stress and is increased in preterm infants (82), is greater in sick vs. stable preterm infants (83), and is increased in patients with chronic obstructive pulmonary disease (86), smokers (87), and workers exposed to traffic exhaust (88).

The question arose whether administration of GRP alone during perinatal transition could lead to histopathological and functional perturbations similar to BPD, even in a clinical setting free of abnormal oxidative stress. To test this hypothesis, we turned to a mouse model, considering that basic molecular mechanisms of lung development have often been explored in mice (89–94).

## Model of Newborn Mice Treated with Exogenous GRP

Extending Koch’s postulates (95) to a non-infectious disease process, we tested whether exogenous GRP would alter lung development in newborn mice. To recapitulate elevated GRP levels shortly after birth, as observed in infants with BPD, we treated newborn mice with bombesin or GRP twice daily from PND 1–3 (11). On Day 14, when alveolarization is normally about half complete, we observed pathological effects similar to BPD induced by bombesin or GRP: alveolar myofibroblast proliferation, increased alveolar wall thickness and diminished alveolarization. Compared with wild-type littermates, bombesin or GRP-treated GRP receptor (GRPR)-null mice (96) had reduced defects in alveolarization, although bombesin-induced interstitial fibrosis was the same as in wild-type littermates. Neuromedin B (NMB) receptor-null (97), and bombesin receptor subtype 3-null (98) mice had the same responses as their wild-type littermates (11). Neither NMB nor a synthetic bombesin receptor type 3 ligand had any effect, consistent with effects of GRP being abrogated in GRPR-null mice. Bombesin/GRP can induce features of BPD, including interstitial fibrosis and diminished alveolarization. GRPR appears to mediate all effects of GRP, but only part of the bombesin effect on alveolarization, suggesting that novel receptors may transduce some effects of amphibian bombesin in newborn lung.

These observations in newborn mice indicate that excessive GRP alone can alter normal lung development, potentially mediating a cascade leading to abnormal pulmonary structure and function weeks to months later. GRP levels are elevated in urine and BAL of asymptomatic smokers (68), who also have elevated oxidative stress markers in urine (99). Maternal smoking is associated with many pediatric lung diseases, including asthma (100). It was hypothesized by Sam Aguayo that GRP could mediate tobacco-related lung diseases (77). We began to explore whether GRP can mediate lung injury due to oxidative stress in older patients, such as that occurring secondary to radiation (RT) exposure.

## GRP and Radiation-Induced Pulmonary Fibrosis

RT-induced lung injury is a clinically relevant model for studying PF in humans, including idiopathic pulmonary fibrosis (IPF). RT produces ROS in target tissues, inducing acute and chronic radiation pneumonitis, and ultimately leading to interstitial fibrosis. In mice and other experimental animals, PF is similar to the human disease caused by environmental exposures or autoimmune diseases, and idiopathic PF. In humans, PF is progressive and irreversible, usually developing over 6–12 months post-RT. The mean survival of patients following the diagnosis of idiopathic PF is 3–5 years. There is no cure for PF except for lung transplantation, which has limited accessibility and has its own set of morbidities. We seek to reverse fibrotic responses in lung by identifying new pathways and bridges preserving organ integrity and homeostasis.

Long-term survivors of childhood malignancies, especially those treated with RT for thoracic tumors, are at a ninefold increased risk of developing PF (101). Post-treatment pulmonary disease is becoming less common with newer modalities of RT therapy such as high-resolution RT and proton beam therapy. In contrast, children undergoing total body irradiation (TBI) prior to bone marrow transplantation frequently develop serious pulmonary sequelae including interstitial fibrosis (102). Like IPF, there is no effective treatment for this post-TBI PF. Similarly, accidental nuclear exposure of children can lead to significant interstitial (restrictive) lung disease that is greater in those individuals exposed to the highest doses of radioactivity (103). Analysis of GRP^+^ PNECs or urine GRP levels in patients post-RT could clarify the disease pathogenesis and potentially set the stage for GRP-blockade treatment to prevent the chronic lung disease in similar clinical settings.

**Figure 2:**
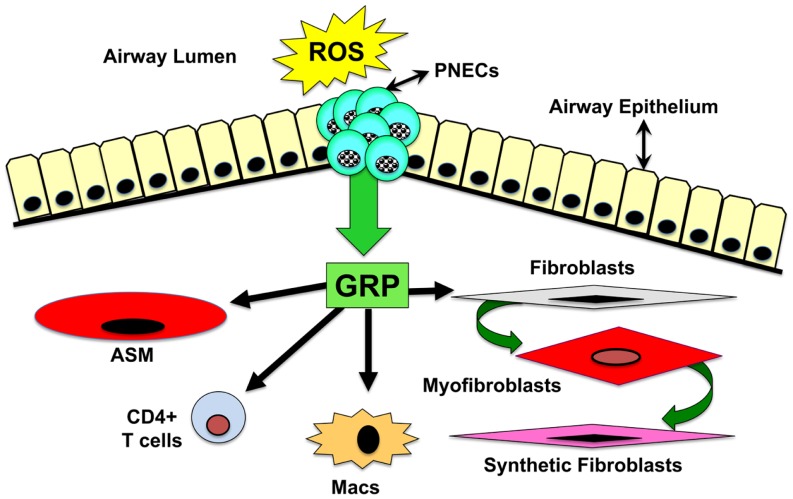
**Schematic drawing of overall hypothesis: mechanisms by which GRP mediates lung injury and fibrosis**. Environmental exposures generating ROS trigger PNECs to secrete GRP, which acts directly on target cells bearing GRPR or NMBR. These target cells include airway smooth muscle cells, macrophages, CD4^+^ T cells, neutrophils, endothelial cells, and pulmonary fibroblasts. Secondary effects could be due to GRP-induced cell differentiation or secretion of cytokines by macrophages and T cells.

Considering that GRP-blockade abrogates pulmonary inflammation and fibrosis in the hyperoxic baboon model of BPD, we sought to determine whether GRP contributes to inflammatory and fibrotic phases of RT induced lung injury. Using a well-characterized mouse model of PF developing ~20 weeks after high-dose thoracic RT (15 Gy) (104), we injected GRP blocking small molecule 77427 1 h after RT then twice weekly for up to 20 weeks (8). Mice given RT plus PBS had increased interstitial CD68^+^ macrophages 4 weeks later and increased GRP^+^/PGP9.5^+^ PNECs 6 weeks later. Ten weeks post-RT, PBS controls had increased pSmad2/3^+^ nuclei indicating active TGFβ signaling. GRP-blockade with 77427 abrogated or significantly diminished CD68^+^, GRP^+^, and pSmad2/3^+^ cells. Twenty weeks post-RT interstitial fibrosis was demonstrated by α-smooth muscle actin (SMA) immunostaining for myofibroblasts (105, 106), which execute organ fibrosis, and also by Masson’s trichrome histochemical staining for interstitial collagen deposition (107, 108). Treatment with 77427 abrogated both interstitial SMA and collagen. Sham mice given 77427 did not differ significantly from PBS controls (8). These observations indicate that GRP-blockade decreases inflammatory and fibrotic responses to RT in mice. Similar to our experiments with hyperoxia and ozone, we propose a general working hypothesis, summarized in Figure 2. Environmental exposures generating ROS trigger PNECs to secrete GRP, which can act directly on target cells bearing cognate receptors, including airway smooth muscle cells (109), macrophages (7), CD4^+^ T cells (7), neutrophils (7, 110), endothelial cells (69), and pulmonary fibroblasts (69). Secondary effects could be due to GRP-induced cell differentiation (46, 50) and/or secretion of cytokines by macrophages and T cells (7, 111). Novel approaches to interrupting GRP signaling could prevent or reverse lung injury and fibrosis caused by RT, hyperoxia, or ozone.

## Potential Relevance of GRP to Other Pediatric Lung Diseases

Additional pediatric lung diseases have been associated with altered numbers of GRP-positive PNECs (17). A large body of work has identified PNECs as airway O_2_ sensors that may function in perinatal adaptation, as detailed above (9, 112). In addition, PNEC and NEB are associated with a stem cell niche that is implicated in airway epithelial regeneration and possibly lung carcinogenesis (17–21). PNEC abnormalities have been described in seemingly unrelated lung diseases, especially PNEC hyperplasia or elevated GRP levels in association with inflammatory lung diseases (113, 114), a few of which will be briefly discussed here. It should be emphasized that increased numbers of PNEC may be due to cell differentiation rather than proliferation (24, 115), and this could represent a general adaptive response to injury or hypoxia. The clinical relevance and precise mechanisms leading to PNEC hyperplasia remain to be explored. Notch family genes (116, 117), human achaete–scute homolog (15, 118), and NeuroD (119) are likely to be involved, but specific signaling defects in patients are unknown.

Idiopathic neuroendocrine cell hyperplasia of infancy (NEHI) has been identified by Robin Deterding as a cause of chronic interstitial lung disease in young children (120–122). The cause of this disorder is unknown. Typically, patients present before 2 years of age with persistent tachypnea, hypoxia, retractions, or respiratory crackles. Lung biopsy findings are non-specific and non-diagnostic, with increased GRP-positive PNECs compared to age-matched controls. Radiographs demonstrate hyperinflation, interstitial markings, and ground-glass densities. Most patients have been treated with O_2_ for long periods of time, but symptoms are generally not eliminated by any medical treatment. Although there has been no mortality in over 5 years of follow-up, a few NEHI patients have improved (120). Thus, NEHI represents a distinct group of pediatric patients with clinical signs and symptoms of interstitial lung disease (120). NEHI may occur in families in some cases (122). However, the abundance of NECs may not fully explain the disease pathogenesis (115).

PNEC hyperplasia has been demonstrated in lungs of infants dying of sudden infant death syndrome (SIDS), possibly secondary to chronic hypoxia in infants at risk (123, 124). Considering that PNECs function as airway O_2_ sensors, Cutz suggested that GRP or another PNEC marker could herald airway chemoreceptor dysfunction as a risk factor for SIDS (125). However, GRP levels are low in SIDS victims, suggesting that another PNEC-derived product could play a role, such as calcitonin gene-related peptide (CGRP) (124). Moreover, parents of SIDS infants have a diminished ventilator response to acute hypercapnia (126), whereas hypercapnia has no effect on PNEC secretion (9).

Cystic fibrosis has also been associated with increased numbers of PNECs immunostaining for GRP, calcitonin, and serotonin (113). CF is a complex lung disease with altered mucus, chronic infection with lung inflammation, and destruction leading to bronchiectasis (127). Urine GRP levels are high postnatally in children with CF, in contrast to the decline in normal infants (76). PNECs express CFTR at the apical membrane, suggesting that NEBs could contribute to CF lung disease, including the early stages before establishment of chronic infection and progressive lung disease (128, 129). Although PNECs, airway innervation, and smooth muscle are altered in Cftr-null mice (130), it remains possible that PNEC abnormalities are secondary to infection and/or inflammation. For instance, NE cell differentiation can be induced by TNFα (131) or other cytokines. At this time, there is no clear-cut evidence for a pivotal role for GRP or PNEC in CF lung disease.

## Time for a Paradigm Shift

Early and excessive GRP secretion is associated with chronic lung disease in infants. With regards to the variable interstitial fibrosis and arrested alveolarization that are characteristic of modern-day BPD, the body of evidence indicates a cause-and-effect relationship: elevated GRP can cause the clinical and pathological hallmarks of BPD in animal models. An NIH observational multicenter clinical investigation of premature infants is currently underway with Judy Voynow and Mike Cotten as PIs, with outcomes including urine levels of GRP and oxidative stress markers. The focus of this collaborative work has now intensified: to determine how transient, early GRP elevation triggers chronic lung disease with fibrosis weeks to months later. Last, but not least, we are actively seeking an optimal approach for GRP-blockade to most effectively prevent BPD in infants and PF in older children and adults.

## Conflict of Interest Statement

The author declares that the research was conducted in the absence of any commercial or financial relationships that could be construed as a potential conflict of interest.
