# Corrigendum: Oxygen, Gastrin-Releasing Peptide, and Pediatric Lung Disease: Life in the Balance

**DOI:** 10.3389/fped.2014.00102

**Published:** 2014-10-02

**Authors:** Mary E. Sunday

**Affiliations:** ^1^Department of Pathology, Duke University Medical Center, Durham, NC, USA

**Keywords:** correction, ozone, gastrin-releasing peptide, lung, injury

Reference # 7 needs to be corrected to: Mary Sunday and Barbara Theriot, unpublished data. We have recently determined that most of the ovalbumin data in the article is unreliable. However, we have successfully validated the ozone data in additional experiments independent of those included in the article, which is the basis for the citation.

## Conflict of Interest Statement

The author declares that the research was conducted in the absence of any commercial or financial relationships that could be construed as a potential conflict of interest.

